# Herlyn-Werner-Wunderlich syndrome: A fertility-sparing approach to a rare mullerian anomaly

**DOI:** 10.5935/1518-0557.20230043

**Published:** 2023

**Authors:** Gabriela Palhano Sifuentes Melo, Thamires Furley Moreira Jandre, Anna Laura Rocha Hermes Vilardo, Roberto de Azevedo Antunes, Daniella Braz Parente, Afrânio Coelho-Oliveira

**Affiliations:** 1 Gynecology Department - University Hospital Clementino Fraga Filho - Federal University of Rio de Janeiro, RJ, Brazil; 2 Fertipraxis - Human Reproduction Center, Rio de Janeiro, RJ, Brazil; 3 Radiology Department of Federal University of Rio de Janeiro, RJ, Brazil

**Keywords:** fertility preservation, uterine anomalies, mullerian ducts, uterine didelphys, solitary kidney

## Abstract

The Herlyn-Werner-Wunderlich syndrome (HWWS) is characterized by the triad of uterus didelphys, obstructed hemivagina, and renal agenesis. The typical clinical presentation involves chronic pelvic pain, dysmenorrhea, and palpable abdominal mass, related to hematocolpos/hematometra. It is a rare disease, with a challenging clinical and radiological diagnosis. Surgery is the definitive treatment. Complications such as endometriosis, infertility and chronic pelvic pain occur more frequently and severely when diagnosis and treatment are delayed. This is a case report of a twelve-year-old patient admitted to the Gynecology Department of the Federal University of Rio de Janeiro’s General Hospital (HUCFF/UFRJ), in March 2021, with progressive symptoms of dysmenorrhea and abdominal distention due to palpable abdominal mass. She had a previous history of congenital solitary kidney. Magnetic Resonance Imaging (MRI) showed a double uterus with hematometra and hematocolpos on the left side, pelvic endometriosis and left renal agenesis. Conservative clinical treatment with inhibition of the hypothalamic-pituitary-ovarian (H-P-O) axis was initiated while a definitive surgical approach was being defined. In June 2022, the patient underwent left hemi-hysterectomy and salpingectomy, achieving full remission of symptoms. Given the rarity of this syndrome and its potential complications, our report aims to familiarize clinicians with it, mostly those who work with children and adolescents, so that more patients have access to early diagnosis and adequate treatment. Consequently, future fertility can be effectively preserved.

## INTRODUCTION

Mullerian anomalies are a rare group of anatomical malformations of the female genital tract. They occur due to alterations during the development and fusion of the Müllerian ducts (or paramesonephric ducts), which normally develop into the uterus, uterine cervix and the two upper thirds of the vagina. Mullerian anomalies are commonly followed by alterations in the development of the Wolff ducts (mesonephric), which later originate the kidneys, justifying the frequent association between Mullerian malformations and urinary tract disorders (Kudela *et al*., 2021).

The Herlyn-Werner-Wunderlich Syndrome (HWWS) is a rare congenital malformation of the female urogenital tract composed of the triad: uterus didelphys, obstructed hemivagina and renal agenesis. Its incidence ranges from 0.1-3.8% in the female population (Kudela *et al*., 2021). In 2007, the term OHVIRA was created - *obstructed hemivagina and renal agenesis* - to describe Mullerian malformations that presented with obstructive uterovaginal syndrome and renal agenesis. The HWWS represents about 77% of OHVIRA cases (Gutiérrez-Montufar *et al*., 2021).

This is the case report of a pubertal patient admitted to the Gynecology Service of the Clementino Fraga Filho University Hospital (HUCFF/UFRJ) in March 2021 with symptoms of abdominal pain beginning right after menarche, associated with a progressive abdominal distension. In her early childhood she was diagnosed with a solitary right kidney, properly located. She was diagnosed with the Herlyn-Werner-Wunderlich syndrome and was submitted to a left hemi-hysterectomy with ipsilateral salpingectomy in June 2022, evolving with complete remission of symptoms.

Our main goal is to reinforce the importance of acknowledging Mullerian malformations in order to maintain an appropriately high index of suspicion, enabling early diagnosis and adequate treatment. Therefore, we are able to improve our patient’s quality of life and reduce the frequency and severity of complications such as infertility.

## CASE DESCRIPTION

This is the case of a 12-year-old patient, brown skinned, born in Rio de Janeiro, Brazil, attending middle school, without previous sexual activity, with a history of moderate to intense dysmenorrhea, which started right after menarche, refractory to common analgesics, associated with progressive increase in abdominal volume. In March 2021, she underwent an abdominal pelvic ultrasonography, which revealed a large complex image, possibly originating from the left ovary. For this reason, she was referred to a quaternary care unit and then admitted to our Clinic at the HUCFF/UFRJ.

The patient had a previous diagnosis of congenital - and anatomically positioned - solitary right kidney, found in a scintigraphy performed at one year of age (March/2010). There were no other relevant morbid or family/social antecedents. She had thelarche and pubarche at age nine, followed by menarche at eleven. Her five first menstrual cycles were regular, with normal duration and flow and after that, progressive dysmenorrhea ensued.

The physical examination revealed adequate Tanner stage (M3P3) and a palpable mass occupying the hypogastrium and left iliac fossa, measuring approximately 10 cm, mobile and painful. Vulvar inspection and speculum exam were normal. Gynecologic bimanual (two-handed) palpation of the uterine corpus found a mass bulging the cul-de-sac on the left side and collapsing the vagina.

A new pelvic US performed at our institution again in March 2021 showed an elongated image with heterogeneous content with debris (10.7x7.6x6.5cm), compatible with blood content, located in the left iliac fossa, laterally adjacent with the right uterine horn. The possibility of left uterine horn with hematometra was considered and the hypothesis of congenital anomaly of the genital tract with consequent obstructive uterovaginal syndrome was raised. We decided to proceed the investigation with a pelvic MRI for diagnostic confirmation and elaboration of a therapeutic approach. In parallel, medroxyprogesterone acetate 150 mg (injectable suspension) was started in order to control signs and symptoms. The patient was well adapted to the method and evolved with secondary amenorrhea. However, she occasionally had episodes of worsening pain, seeking emergency medical care for parenteral analgesia.

**Figure 1:**
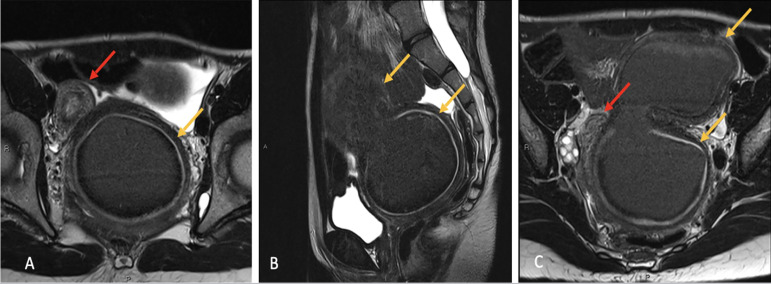
A. Axial T2-weighted MRI image shows a uterus didelphys with two separate uterine cavities (arrows). The left uterine cavity is distended by blood products due to presumed cervix-vaginal atresia on the left side. B. Sagittal T2-weighted MRI image shows the left uterine cavity distended by blood products. C. Axial T2-weighted MRI image shows a uterus didelphys with two separate uterine cavities (arrows).

A pelvic MRI was performed in May 2021, demonstrating uterine duplicity with significant hematometra and hematocolpos on the left side (Figure 1A, 1B, 1C) left renal agenesis, ligament and peritoneal endometriosis. However, the method alone could not evaluate the presence of a vaginal septum. Therefore, in order to better elucidate the anatomical variation, a diagnostic video-hysteroscopy was performed in August 2021, finding a patent right uterine cervix with a small right uterine cavity. Left uterine cervix and vaginal septum were not visualized. At that moment, we concluded she had a single right patent vagina, presumed cervix-vaginal atresia on the left side, two hemi-uterus without communication between them, leading to hematocolpos and hematometra, in addition to a previously known left renal agenesis. In view of these findings, the diagnosis of a variant of the Herlyn-Werner-Wunderlich syndrome (HWWH) was established.

Considering the MRI and hysteroscopic findings, a surgical approach was planned, initially via vaginoscopy, with the aim to drain the left hematocolpos and hematometra. If vaginal access was not feasible, a laparotomy approach was suggested at the same surgical time for left hemi hysterectomy. The surgical plan was presented to the patient and her guardians, who understood and approved it, signing an informed consent form.

**Figure 2:**
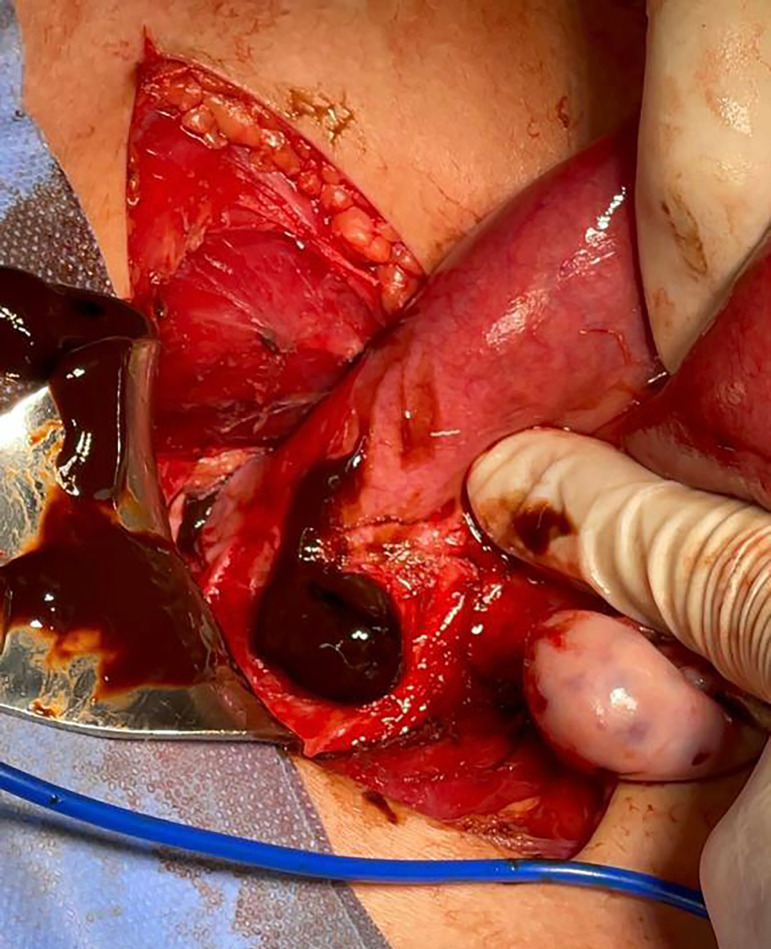
Drainage of voluminous content of blood from previous hematometra and hematocolpos before left hemi - hysterectomy.

The patient underwent surgery in July 2022. After an unsuccessful attempt at vaginoscopy under sedation, laparotomy was performed with left hemi hysterotomy followed by drainage of hematometra and hematocolpos (Figure 2). Posteriorly, we completed the procedure with a hemi hysterectomy and salpingectomy. The anatomopathological analysis revealed a left hemi-uterus with extensive stromal decidualization, an atretic left cervix without an ectocervical component and a left uterine tube with hematosalpinx. The postoperative period followed without complications and the patient was discharged within 48 hours. She evolved with complete remission of symptoms and is currently undergoing clinical follow-up at our Gynecology clinic.

## DISCUSSION

The Herlyn-Werner-Wunderlich syndrome (HWWS) is a rare mullerian anomaly, characterized by the triad: uterus didelphys, obstructed hemivagina and ipsilateral renal agenesis. It was first described in 1922 (Gutiérrez-Montufar *et al*., 2021; Jomaa *et al*., 2021; Vo Nhu *et al.*, 2021), but it was only named after publications by Herlyn and Werner in 1971 and Wunderlich in 1976 (Gutiérrez-Montufar *et al*., 2021). In 2007, a new term was suggested to describe the association between obstructive uterovaginal syndrome and renal abnormalities, the OHVIRA syndrome (obstructed hemivagina and renal agenesis), which allows the inclusion of its multiple and heterogeneous clinical presentations (Gutiérrez-Montufar *et al*., 2021; Vo Nhu *et al*., 2021). In view of the large spectrum of anatomical variations and their subclassifications, in 2021 a new and more descriptive classification was proposed by the American Society for Reproductive Medicine (ASRM), which aimed to facilitate the diagnosis and clinical-surgical decision (Pfeifer *et al*., 2021).

Several combinations of these malformations are currently described and since the 80’s there have been various attempts to categorize them (Tuna *et al*., 2019). However, to date, none of the proposed classifications has been sufficient to fully describe all the possible existing variations (Pfeifer *et al*., 2021). This reflects the great difficulty in diagnosing and managing patients with these anomalies, which justifies the recurrent delay in diagnosis and frequent complications, such as endometriosis and infertility.

The HWWS corresponds to 77% OHVIRA syndrome cases (Gutiérrez-Montufar *et al*., 2021) and its incidence varies around 0.1-3.8% in the female population (Kudela e*t al.*, 2021; Vo Nhu *et al*., 2021; Girardi Fachin *et al*., 2019). The advance of imaging methods facilitated the diagnosis of congenital Mullerian anomalies, including the HWWS (Gutiérrez-Montufar *et al*., 2021; Kudela *et al*., 2021).

The coexisting disorders of the urinary and reproductive systems in a female patient suggests a simultaneous alteration in the embryonic development of the Wolff (mesonephric) and Müller (paramesonephric) ducts (Vázquez Gómez *et al*., 2021; Vo Nhu *et al*., 2021). In females, the mesonephric ducts regress while the paramesonephric ducts fuse at their distal end to yield the uterus, cervix, and upper two-thirds of the vagina; and persist uncombined near its proximal part to generate the fallopian tubes (Jomaa *et al*., 2021; Sharma *et al.*, 2016). The embryologic formation of the reproductive and urinary tract systems occur simultaneously and while the kidneys originate from the wolffian ducts the lower third of the vagina derives from the urogenital sinus (Vo Nhu *et al.*, 2021). It is important to note that the ovaries and fallopian tubes are not affected in these conditions, since they go through a distinct developmental process (Jomaa *et al*., 2021).

The main cause of congenital Müllerian or paramesonephric duct anomalies is defective fusion (vertical or horizontal) or septal resorption failure (Vo Nhu *et al*., 2021). If the mullerian ducts do not fuse, the uterine horns, cervix, and endometrial cavities appear separately (Jomaa *et al*., 2021; Sharma *et al.*, 2016). On the other hand, unilateral renal agenesis comes from an abnormality of the mesonephric ducts (Gutiérrez-Montufar *et al*., 2021). Therefore, in the face of female patients presenting with urinary system malformations, it is mandatory to investigate associations with genital tract anomalies, and vice versa (Kudela *et al*., 2021).

Regarding the heterogeneities in its clinical presentation, variations in urinary disorders stand out, renal agenesis being the most common, although there are reports of multicystic kidney, dysplastic kidney, horseshoe kidney, pelvic kidney and ectopic ureters (Kudela *et al.*, 2021; Jomaa *et al.*, 2021). On the other hand, among the uterine variations, most patients present with a didelphys uterus, but bicornuate, septate or single uterus have also been reported (Kudela *et al.*, 2021). The right side is usually the most affected.

The multiple anatomical presentations justify the great variability of reported symptoms, along with their frequency and intensity, modifying the amount of time required to establish a final diagnosis and the ideal surgical approach for each case. The main related variation is the type of vaginal obstruction, which can be complete or incomplete (Vo Nhu *et al*., 2021; Zhu *et al*., 2015). Thus, Lan Zu *et al.* (2015) proposed a subclassification of HWWS into type 1 and type 2, according to the type of obstruction (Zhu *et al*., 2015).

In type 1, there is a hemivagina with complete obstruction, which is subdivided into blind hemivagina or cervicovaginal atresia. In the case of blind hemivagina, the vaginal septum results in a complete obstruction, so that there is no communication between the two hemi uteri or between the vaginas. Therefore, the obstructed side develops hematocolpos, which may evolve with hematometra and hematosalpinx. In these cases, the age of onset of symptoms is earlier with a short time from menarche (Girardi Fachin *et al*., 2019; Zhu *et al*., 2015; Nishu *et al*., 2019).

Endometriosis is the most frequent complication (Girardi Fachin *et al*., 2019; Zhu *et al*., 2015), but pyocolpos, pyosalpinx and pelvic adhesions are also associated (Zhu *et al.*, 2015). In the subtype that courses with cervicovaginal atresia, without communication with the uteri, there are rudimentary uterine cervix and hemivagina, which therefore also maintains complete obstruction, coursing with a similar clinical presentation and the later complications described above.

Type 2, characterized by a hemivagina with incomplete obstruction, on the other hand, is subdivided into partial reabsorption of the vaginal septum and the presence of uterine communication. In cases where there is partial reabsorption of the septum, there is communication between the two vaginas, so that, despite the uteri being isolated from each other, a drainage orifice is created. These patients have a later age of onset. The attack often comes years after menarche and the symptoms are more tolerable. However, the presence of this communication favors the occurrence of purulent or bloody vaginal discharge and ascending genital system infections. In cases where there is uterine communication, menstruation flows from one hemiuterus to the contralateral one, even in the presence of obstructed hemivagina, reducing the accumulation of menstrual flow and delaying both the age of diagnosis and the complications associated with the syndrome (Girardi Fachin *et al*., 2019).

The 2021 ASRM classification is based on nine major findings: mullerian agenesis; cervical agenesis; unicornuate uterus; uterus didelphys; bicornuate uterus; septate uterus; longitudinal vaginal septum; transverse vaginal septum and complex anomalies. These findings may undergo variations and are often related to each other, composing the various existing anomalies. In addition to these, there are also several anomalies in the urinary tract (Pfeifer *et al.*, 2021).

Considering all the possible anatomical variations, it is understandable that most patients remain asymptomatic until puberty (Nishu *et al*., 2019). Commonly, after menarche, pelvic pain, dysmenorrhea and menstrual alterations appear, worsening at each menstrual cycle, associated with an increase in abdominal volume and a palpable abdominal mass (Gutiérrez-Montufar *et al*., 2021; Jomaa *et al.*, 2021; Vo Nhu *et al*., 2021; Nishu *et al*., 2019). It is common to seek emergency services in the context of pain exacerbation, simulating acute abdominal conditions. Most of the diagnoses are made during this period, already in adolescence (Vázquez Gómez *et al*., 2021; Vo Nhu *et al*., 2021; Girardi Fachin *et al*., 2019). Other symptoms reported were dysuria and a prolapsed mass via the vagina. Urinary retention is a rare form of presentation (Gutiérrez-Montufar *et al*., 2021).

In pre-pubertal patients, the main finding is a palpable abdominal mass (Gutiérrez-Montufar *et al.*, 2021; Sharma *et al.*, 2016). Although uncommon, this malformation can be diagnosed in the neonatal period before any clinical manifestation, usually after a prenatal US diagnosis of renal agenesis. Hydrocolpos can also be detected in the neonatal period or even in the prenatal period, being reported as early as the 25^th^ week of pregnancy. The most common finding in the neonatal period is a soft vulvar mass. However, perineal examination is difficult at this age, making the differential diagnosis with imperforate hymen a challenge (Tuna *et al*., 2019).

There are few cases of patients diagnosed in the adult stage (Kudela *et al*., 2021), with symptoms such as dysmenorrhea and chronic pelvic pain having been reported (Gutiérrez-Montufar *et al*., 2021; Jomaa *et al*., 2021). Endometriosis was identified in approximately 10.3% (Gutiérrez-Montufar *et al*., 2021) to 13.6% (Kudela *et al*., 2021) of the cases, being the theory of “retrograde menstruation” the pathophysiological basis (Jomaa *et al*., 2021). Vaginal discharge, pyocolpus, dyspareunia, and infertility have also been described (Gutiérrez-Montufar *et al*., 2021; Kudela *et al*., 2021; Jomaa *et al.*, 2021). Abnormal uterine bleeding is less frequent, but has also been reported, mainly in cases of partial vaginal septum. There are also reports of diagnosis performed during pregnancy, after bleeding in the first trimester (Gutiérrez-Montufar *et al.*, 2021).

Delay in the diagnosis of HWWS is common due to factors such as the assistant physicians’ lack of knowledge, the presence of a regular menstrual cycle due to an unobstructed type of malformation and improvement of symptoms with prescription of common analgesics, NSAIDs and hormonal contraceptives (Jomaa *et al*., 2021; Girardi Fachin *et al*., 2019). Chronic pelvic pain syndrome, endometriosis, infertility and habitual spontaneous abortion are frequent complications and the severity of these complications is directly related to this delay (Girardi Fachin *et al*., 2019; Nishu *et al*., 2019).

HWWS diagnosis is made by imaging tests, the most frequently used are pelvic US and MRI (Gutiérrez-Montufar *et al*., 2021; Kudela *et al*., 2021). The gold standard, however, is direct visualization via laparoscopy, being reserved for selected cases, where there is surgical indication, as it is an invasive method (Gutiérrez-Montufar *et al.*, 2021). Other complementary tests such as hysteroscopy may be indicated, but they are not essential and are often insufficient for the diagnosis.

Abdominopelvic US is a good initial choice due to its greater accessibility, lower cost and lack of associated adverse effects (Vo Nhu *et al*., 2021; Sharma *et al*., 2016). This method may be enough to establish the diagnosis and determine treatment onset (Jomaa *et al.*, 2021). It can identify uterine anomalies, such as uterus didelphys and urinary alterations, such as renal agenesis. There may also be hematocolpos, hematometra or hydrosalpinx. However, it is not an adequate method for evaluating the vagina and vaginal septum (Gutiérrez-Montufar *et al*., 2021; Kudela *et al*., 2021; Jomaa *et al*., 2021; Vo Nhu *et al*., 2021).

MRI is more expensive and less accessible, but it is more accurate in diagnosing uterine malformations (Gutiérrez-Montufar *et al.*, 2021; Kudela *et al*., 2021; Girardi Fachin *et al.*, 2019). This method is adequate for cases in which the US was not sufficient to assess the vaginal septum and to better evaluate the malformation itself. It also enables the assessment of associated complications, such as endometriosis and pelvic adhesions (Vo Nhu *et al*., 2021; Girardi Fachin *et al.*, 2019; Nishu *et al*., 2019). It is especially recommended if surgical treatment is indicated (Kudela *et al*., 2021; Jomaa *et al*., 2021). Computed tomography (CT) can be an alternative exam; however, it is less accurate for the evaluation of pelvic structures, in addition to exposing the patient to ionizing radiation and, therefore, it should be used only in exceptional cases, when MRI is not available (Girardi Fachin *et al.*, 2019).

The definitive treatment for the syndrome is surgical, seeking not only to relieve symptoms, but also to improve reproductive outcomes and reduce long-term complications (Jomaa *et al*., 2021; Girardi Fachin *et al*., 2019). Timing of surgery is still controversial in the literature. Some authors advocate conservative management until puberty, while others suggest that, upon diagnosis, surgery should be promptly indicated.

Therefore, safe conservative clinical management until puberty is acceptable. Possible hydrocolpos that present after birth usually resolve spontaneously in the first months of life, when circulating maternal estrogen levels decline. If there are recurrent urinary tract infections or urinary incontinence in childhood, or even large vaginal masses, drainage procedures or early vaginal septectomy may be indicated (Kudela *et al.*, 2021). The surgical approach depends on the type of anatomical variation. In general, the access can be via vaginoscopy or laparoscopy/laparotomy, with satisfactory results in both cases (Jomaa *et al.*, 2021).

In cases where there is a vaginal septum, it can be resected via vaginoscopy with drainage of the hematocolpos (Kudela *et al*., 2021; Jomaa *et al*., 2021; Vo Nhu *et al.*, 2021) and preservation of the hemiuterus (Girardi Fachin *et al.*, 2019), being sufficient as treatment in 86.5% of cases (Kudela *et al*., 2021). There is no need to suture the vaginal wall after resection of the septum, and it may heal by second intention (Kudela *et al.*, 2021).

On the other hand, in the presence of HWWS associated with cervicovaginal atresia, a more complex approach is necessary, since vaginal access may not be possible, requiring a hemihysterectomy. Ipsilateral salpingo-oophorectomy is a possibility (Kudela *et al.*, 2021; Vo Nhu et *al.*, 2021). The preferred approach is laparoscopic, as it is less invasive, but laparotomy can be used if laparoscopy is not available, with acceptable results (Jomaa *et al.,* 2021; Sharma *et al.,* 2016).

One of the main concerns of patients and their families is future fertility. Based on the analyzed studies, the obstetric results after resection of the vaginal septum, were satisfactory in most cases (Kudela *et al.*, 2021). However, these pregnancies are conditioned to more frequent complications, such as spontaneous abortions, ectopic pregnancy, preterm delivery and cesarean delivery (Kudela *et al.*, 2021; Jomaa *et al.*, 2021).

The work of Lan Zu et al. of 2015 found that the percentage of patients wishing to conceive who had at least one pregnancy was around 64-95% (Zhu *et al.*, 2015); and of these, most pregnancies occurred on the side, contralateral to the surgical approach (Kudela *et al.*, 2021). However, it is possible that it occurs on the affected side in about 52.9% of the cases, after resection of the vaginal septum (Salastekar *et al.*, 2019). Girardi Fachin et al. (2019) retrospectively analyzed 36 patients with HWWS for a period of 30 years and concluded that, after treatment, 87% of the patients with a desire to become pregnant had a successful pregnancy, with a total rate of 77% live births (15% premature, 62% at term).

Regarding endometriosis and chronic pelvic pain, surgical treatment with cytoreductive laparoscopic surgery was recently considered, without significant obstetric outcomes, considering the complications inherent to the procedure (adhesions, fibrosis and tubal obstruction). It was therefore concluded that laparoscopy should only be performed in patients, refractory to medical treatment and with evidence of deep/invasive disease (Kudela *et al.*, 2021).

## CONCLUSION

The Herlyn-Werner-Wunderlich syndrome should be known by clinicians who are involved with child and youth health care in order to improve these patients’ quality of life. The diagnosis is clinical and radiological, and should be confirmed as early as possible in order to reduce further complications. Future fertility is one of the main concerns, which can be successfully preserved, even though obstetric complications occur more often. The definitive treatment is surgical, although the best timing is still under debate. The approach must be individualized according to the anatomical variation presented by the patient. A multidisciplinary approach involving clinicians, surgeons and radiologists is essential to achieve the best outcomes.
